# Optimizing autologous hematopoietic stem cell transplantation for acute leukemia

**DOI:** 10.1002/sctm.21-0176

**Published:** 2021-11-01

**Authors:** Aiming Pang, Yingying Huo, Biao Shen, Yawei Zheng, Erlie Jiang, Sizhou Feng, Mingzhe Han

**Affiliations:** ^1^ State Key Laboratory of Experimental Hematology, National Clinical Research Center for Blood Diseases, Hematopoietic Stem Cell Transplantation Center, Institute of Hematology and Blood Diseases Hospital, Chinese Academy of Medical Sciences and Peking Union Medical College Tianjin People's Republic of China

**Keywords:** acute leukemia, autologous hematopoietic stem cell transplantation, China, recommendations

## Abstract

Autologous hematopoietic stem cell transplantation (ASCT) remains an important postremission treatment for acute leukemia (AL). It is known that some prognostic factors, such as age, cytogenetic and molecular risk stratification, and minimal residual disease (MRD) status, are closely related to clinical outcomes following ASCT. Moreover, there are multiple measurements, including pretransplant treatment, stem cell mobilization and collection, conditioning regimens, and maintenance treatment after transplantation, that can affect prognosis after ASCT. Our clinical practice of ASCT should be better standardized to further improve patient outcomes. This review outlines optimization and quality control measures for ASCT developed at the Institute of Hematology and Blood Diseases Hospital of the Chinese Academy of Medical Sciences, the first established and largest autologous stem cell transplant center in China. These measures will enhance the development of best practices and strategies for AL ASCT therapies, thereby improving patient outcomes.

## Graphical abstract

Schema for autologous hematopoietic stem cell transplantation in patients with acute leukemia at the Institute of Hematology and Blood Diseases Hospital of Chinese Academy of Medical Sciences.

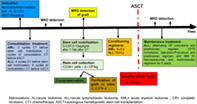

**Significance statement**

Autologous hematopoietic stem cell transplantation (ASCT) is recommended for favorable‐risk acute myeloid leukemia patients in first complete remission (CR1) with negative minimal residual disease and is an important option for intermediate‐risk patients in CR1. It is also a treatment option for the patients with Philadelphia‐positive (Ph^+^) acute lymphocytic leukemia (ALL) with a complete molecular response within 3 months, maintained up to transplantation. The consolidation chemotherapy and intrathecal chemotherapy should be given before ASCT. The authors recommend maintenance chemotherapy for ALL after ASCT and tyrosine kinase inhibitors are also in the list of maintenance regimens for Ph^+^ ALL after ASCT.

## 1 INTRODUCTION

Autologous hematopoietic stem cell transplantation (ASCT) is an important postremission treatment for acute leukemia (AL). Professor Norbert‐Claude Gorin performed the first ASCT for an acute myeloid leukemia (AML) patient in 1976, establishing a paradigm for treating AL. In 1986, the first ASCT in China was carried out at the Institute of Hematology (IH) by Professor Wenwei Yan, who then went on to perform the largest number of ASCT cases. To date, as allogeneic hematopoietic stem cell transplantation (HSCT) has become widely available, ASCT for adult patients with AL has declined but it still considered as an effective treatment for favorable and intermediate AML patients in first complete remission (CR1), as well as acute lymphocytic leukemia (ALL) with minimal residual disease (MRD)‐negative status, including Philadelphia‐positive (Ph^+^) ALL. In short, ASCT produces satisfying overall survival (OS) and leukemia‐free survival (LFS) in adult AL patients. Here, we review and discuss our historical data and recommendations for ASCT based on the experience at IH.

## 2 BASIC CONCEPTS OF HEMATOPOIETIC STEM CELLS

From 1961 to 1963, Till and McCulloch first observed colony‐forming units in the spleen of transplanted mice,^1^, ^2^ and subsequently defined functional hematopoietic stem cells (HSCs). In the conventional model of the hematopoietic differentiation tree, HSC lie at the top of the hierarchy and differentiate to common lymphoid progenitors (CLP) and common myeloid progenitors (CMP), thus giving rise to all blood cell types.^3^ Stem cells exhibit five minimal functional states, namely *s*elf‐renewal, *m*ultilineage differentiation, *a*poptosis, *r*esting mode, and *t*rafficking, which constitute the so‐called “SMART” model for maintaining stem cell homeostasis in vivo.^4^, ^5^ The mechanisms underlying HSC homeostasis involve endogenous and exogenous regulations. Endogenous regulation represents several classical signal pathways (such as the Notch and Wnt signaling pathways) and key transcriptional regulators,^6^, ^7^ whereas exogenous regulation is more inclined to refer to the regulatory unit, defined as the bone marrow (BM) niche. The components of the BM niche include cell constituents, cell factors, and metabolites such as reactive oxygen species.^8^, ^9^, ^10^, ^11^

## 3 INDICATIONS

Extensive previous studies have evaluated the effects of ASCT for AL patients, especially for those with favorable risk and intermediate risk in CR1. Most of them acknowledging the superiority of ASCT over chemotherapy and allogeneic HSCT (allo‐HSCT) emphasized relapse, survival, and safety.

### 3.1 Acute myeloid leukemia

In an early prospective randomized study, AML patients who achieved CR after induction treatment with daunorubicin and cytarabine received a first course of intensive consolidation chemotherapy, combining intermediate‐dose cytarabine and amsacrine. Then, these patients were randomly assigned to undergo ASCT or a second course of intensive chemotherapy, and ASCT resulted in better LFS than intensive consolidation.^12^ Subsequently, another prospective randomized phase 3 trial evaluated the outcomes of 258 ASCT vs 259 chemotherapy patients who were in CR1 and received 2 cycles of intensive chemotherapy.^13^ The results further demonstrated that ASCT, compared with chemotherapy, remarkably reduced the relapse rate (58% vs 70% at 5 years, *P* = .02) and improved LFS (38% vs 29% at 5 years, *P* = .065).^13^ OS was similar between the two treatment arms (44% vs 41% at 5 years, *P* = .860), which might be attributable to salvage chemotherapy and HSCT for relapse patients on the chemotherapy arm.^13^ Similar results were reported in other studies.^14^, ^15^ For example, a total of 465 patients aged 40 to 60 years in CR1 were given chemotherapy or ASCT and the results revealed that ASCT was associated with a reduced risk of relapse (hazard ratio (HR) 0.66, 95% confidence interval [CI] 0.50‐0.87, *P* = .003) and improved LFS (HR 0.69, 95% CI 0.53‐0.90, *P* = .006).^14^ In addition, nonrelapse mortality (NRM) was not significantly different when comparing ASCT and chemotherapy (HR 2.13, 95% CI 0.69‐6.56, *P* = .180), supporting the acceptable safety of ASCT.^14^ Many studies have compared the clinical outcomes of ASCT with those of allo‐HSCT to determine the superior treatment option.^12^, ^14^, ^15^, ^16^, ^17^, ^18^, ^19^

**FIGURE 1:**
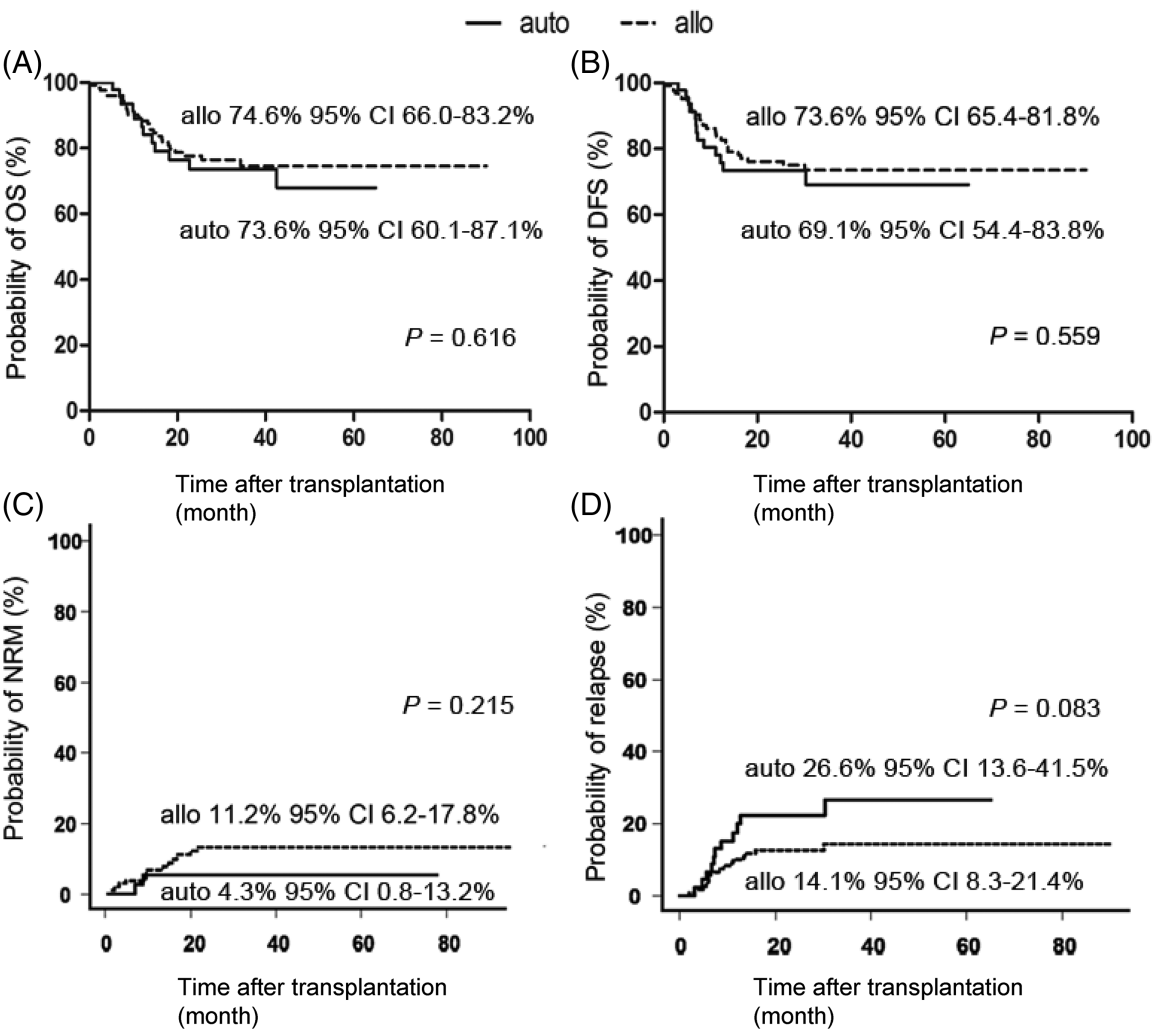
Prognosis of acute myeloid leukemia (AML) patients after auto‐ and allo‐stem cell transplantation (SCT).^17^ A, The overall survival (OS); B, disease‐free survival (DFS); C, nonrelapse mortality (NRM); D, leukemia relapse, in auto group (n = 46, solid line) and allo group (n = 126, broken line)

**FIGURE 2:**
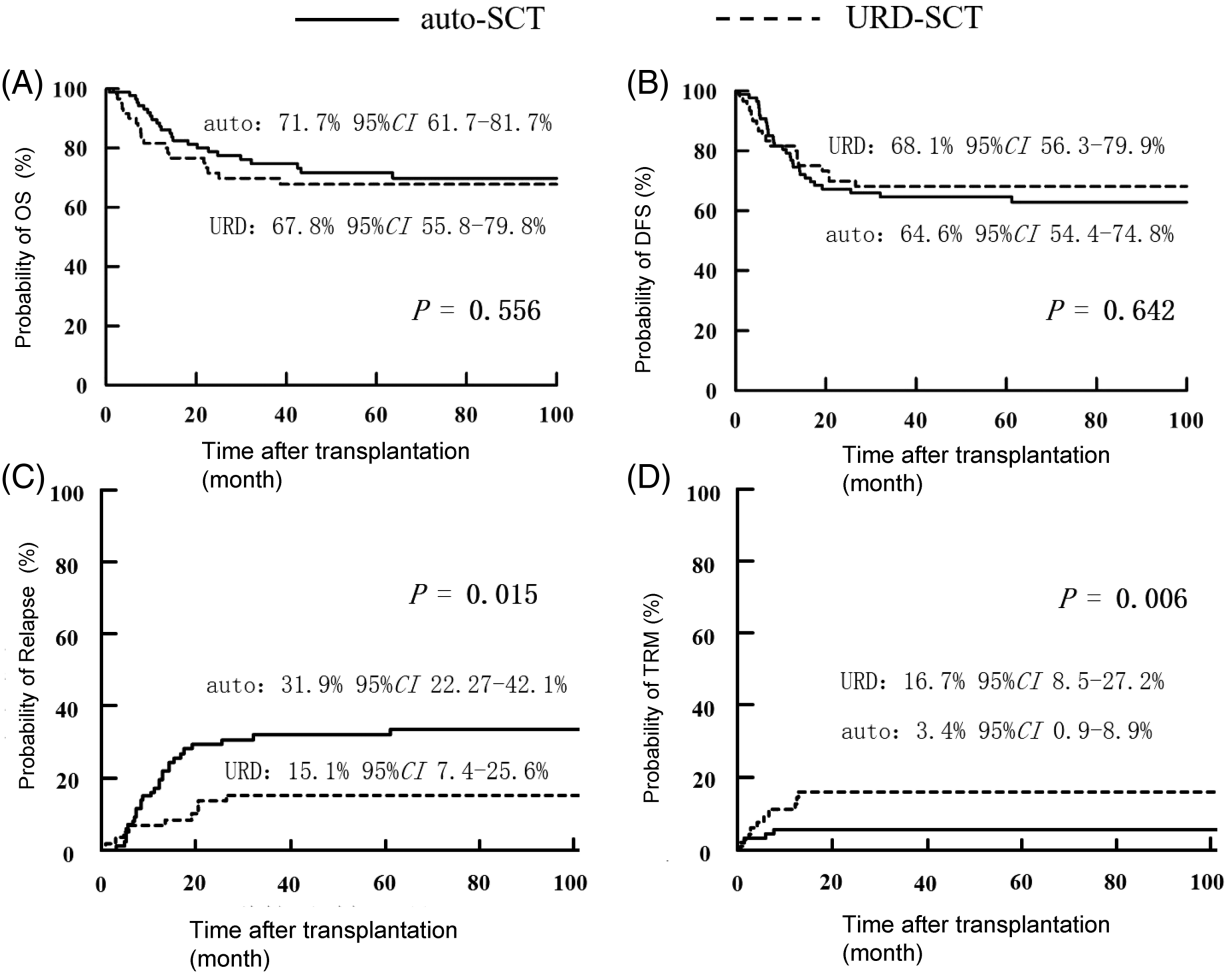
Prognosis of acute myeloid leukemia (AML) patients after auto‐ and unrelated donor (URD)‐SCT.^22^ A, The overall survival (OS); B, disease‐free survival (DFS); C, relapse; D, transplant‐related mortality (TRM), in auto group (n = 87, solid line) and allo group (n = 60, broken line)

MRD status before ASCT is an independent prognostic factor for both OS and RFS after ASCT.^20^ Our data show that MRD detection after one course of consolidation chemotherapy was an independent prognostic factor for 3‐year OS (83.1% vs 19.0%; *P* = .006) and disease‐free survival (DFS; 73.9% vs 14.2%; *P* = .049) in patients with AML who underwent ASCT in CR1.^21^ Furthermore, we identified cytogenetic risk as an independent prognostic factor for survival outcomes, with decreasing OS and DFS as risk increased.^21^ It is also demonstrated that ASCT and allo‐HSCT offered comparable DFS for patients who were in favorable or intermediate risk and tested MRD (−) after one course of consolidation (*P* = .270), otherwise ASCT was inferior due to the increased risk of leukemia relapse (Figure 1). Mizutani et al conducted a retrospective study involving 350 AML patients with normal cytogenetics in CR1, 177 ASCT cases and 173 matched unrelated donor (MUD) HSCT ones, and observed comparable OS and LFS between the two groups.^19^ In another risk‐stratified outcome analysis, AML patients in CR1 were assigned to favorable risk group, intermediate‐1 group or intermediate‐2 group according to the European Leukemia Net classification.^18^ It found that ASCT was superior to MUD‐HSCT in the favorable risk group, with a lower NRM and a better OS, while MUD‐HSCT was associated with a better LFS and OS in the intermediate‐1 group. In the intermediate‐2 group, the advantage of lower NRM in ASCT group may have been offset by the advantage of lower relapse rate in MUD‐HSCT group, as the two groups showed similar OS and LFS.^18^ Yao et al compared the difference between ASCT and MRD‐HSCT for adults with primary AML in CR1. After a median follow‐up of 53.8 (0.8‐127.8) months, patients in the two groups demonstrated comparable OS and LFS 5 years after the transplant (71.7% vs 67.8%, *P* = .556; 64.6% vs 68.1%, *P* = .642, respectively), and there was no significant difference between the two groups^22^ (Figure 2). A retrospective study compared ASCT and haploidentical HSCT (haplo‐HSCT) in 196 AML patients with favorable and intermediate risk in CR1. The results indicated a significantly lower NRM for ASCT patients.^20^ However, the superiority of ASCT was impaired by a higher incidence of relapse, which resulted in a similar 3‐year OS and RFS.^20^ In the subgroup analysis of intermediate‐risk patients, haplo‐HSCT yielded better OS due to higher incidence of relapse in ASCT patients.^20^

Collectively, ASCT and allo‐HSCT yielded similar outcomes regarding OS and RFS. However, the potential impact of acute and chronic graft vs host disease caused by allo‐HSCT on the quality of life of patients was not considered in these studies. Furthermore, regarding the burden of hospitalization and challenging supportive treatment for MUD‐HSCT and haplo‐HSCT, ASCT is recommended for favorable‐risk AML patients in CR1 and is an attractive option for intermediate‐risk patients in CR1. In addition, as a result of induction chemotherapy utilizing all‐trans retinoic acid and arsenic trioxide, acute promyelocytic leukemia (APL) has become a highly curable disease. Besides, there is a role for ASCT in CR2 patients with APL to achieve a cure.^23^ A study on the European Society for Blood and Marrow Transplantation showed that ASCT resulted in better survival outcomes for APL in CR2. The LFS and OS in ASCT patients were 74.5% (95% CI 69‐79.2) and 82.4% (95% CI 77.3‐86.5) compared with those of 54.7% (95% CI 47.5‐61.3) (*P* = .001) and 64.3% (95% CI 57.2‐70.6) in allo‐HSCT patients, respectively (*P* = 0.001 and *P* = 0.001).^24^

### 3.2 Acute lymphocytic leukemia

There were studies on ASCT in adult ALL patients including B‐cell acute lymphoblastic leukemia (B‐ALL) and T‐cell acute lymphoblastic leukemia (T‐ALL). A recent study on MRD after first induction treatment (MRD1) in adult ALL patients treated with ASCT showed that the proportion of high‐risk immunophenotype (pro‐B, pro‐T, pre‐T, mature T) was significantly higher in MRD1‐positive patients than that in MRD1‐negative patients (34.6% vs 14.5%, *P* = .038). Positive MRD1 and high‐risk immunophenotype were risk factors for LFS (HR = 3.986, 95% CI 1.813‐8.764, *P* = .001; HR = 2.981, 95% CI 1.373‐6.473, *P* = .006). ASCT could not reverse the poor prognosis of MRD1‐positive patients. ASCT treatment is optional for MRD1‐negative patients who maintained MRD1 negative during intensive therapy.^25^ In ALL patients who achieved CR1 with induction therapy and received at least 4 cycles of early‐stage intensive consolidation chemotherapy, ASCT was demonstrated to be a better postremission option compared with chemotherapy.^26^ The ASCT group had higher LFS and OS after 3 and 5 years. However, the difference was not statistically significant at 1 year, which reminds us of the importance of long‐term follow‐ups.^26^

To compare the outcomes of ASCT and allo‐HSCT in ALL patients, 50 patients with negative MRD receiving ASCT and 56 patients receiving allo‐HSCT were retrospectively analyzed.^27^ There was no significant difference between the two groups in the probability of 3‐year OS (74.1% vs 55.1%) and LFS (63.7% vs 53.7%), indicating acceptable efficacy of ASCT in ALL patients with negative MRD.^27^

Another study reported a significantly lower NRM and a better OS of ASCT compared with those of haplo‐HSCT in 159 ALL patients with CR (2‐year NRM, 3% vs 30%, *P* < 10^−5^; 2‐year OS, 66% vs 40%, *P* = .010). There was no significant difference in the 2‐year LFS, OS and relapse rate between the two groups.^28^ Further subgroup analysis found that Ph^+^ ALL patients in CR1 benefited more from ASCT compared with haplo‐HSCT. The 2‐year LFS, OS, and NRM were 60% vs 26% (*P* = .005), 76% vs 26% (*P* = .001) and 4% vs 43% (*P* < .001) in the ASCT vs haplo‐HSCT groups, respectively.^28^ These findings indicated that ASCT could be considered as an effective consolidation therapy in CR1 ALL patients at standard risk or with Philadelphia chromosome.

High‐risk patients are not suitable for ASCT. An observational study at IH showed that patients with Ph‐negative high‐risk B ALL had significantly lower OS and LFS in comparison to standard‐risk B‐ALL patients (3‐year OS: 46.1% vs 77.6%, *P* = .007; 3‐year LFS: 39.1% vs 76.3%, *P* = .001).^29^ Positive MRD before ASCT (MRD ≥0.01%) also indicated worse outcomes and the further demand for allo‐HSCT.^29^ However, it is noteworthy that high risk was not an absolute contraindication. The development of pre‐HSCT purification and post‐HSCT maintenance therapy can improve the prognosis of ASCT patients. One study reported a 73% 3‐year LFS compared to 42.2% for nontreated ASCT, and 50.9% for allo‐HSCT in adult patients with T‐cell ALL (*P* < .05).^29^ Thus, for those CR patients with no available donor, ASCT combined with graft purification and maintenance therapy can be effectively used as an alternative treatment.

**FIGURE 3:**
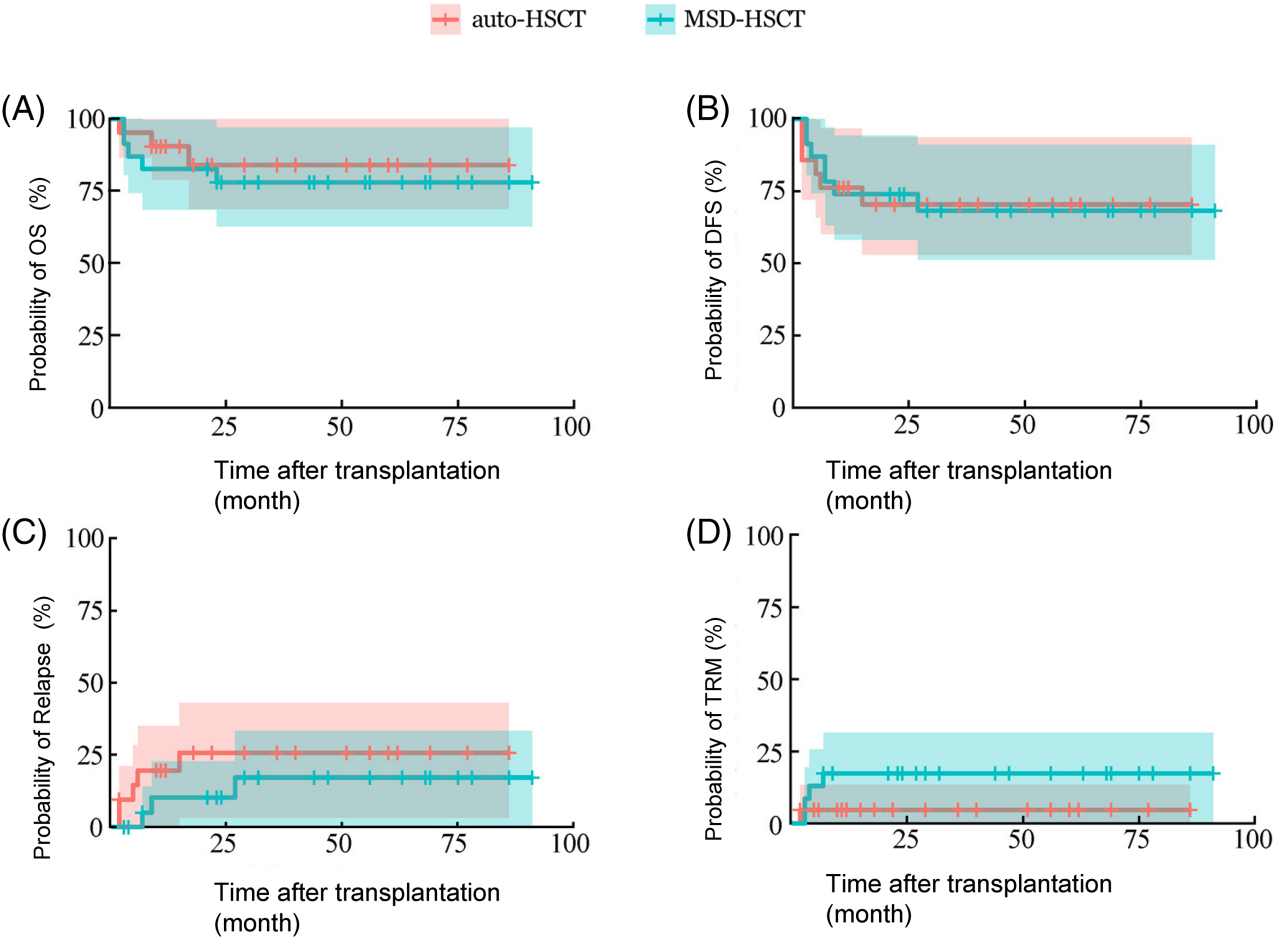
Prognosis of Philadelphia‐positive (Ph^+^) acute lymphocytic leukemia (ALL) patients after auto‐ and matched sibling donor (MSD)‐SCT.^30^ A, The overall survival (OS); B, disease‐free survival (DFS); C, relapse; D, transplant‐related mortality (TRM), in auto group (n = 31, solid line) and allo group (n = 47, broken line)

Lyu et al compared the efficacy of ASCT and MSD‐HSCT in Ph^+^ ALL patients and found that OS, LFS, and NRM at 3 years were not significantly different between the two groups (*P* > .05).^30^ They also reported that there is no significant difference in terms of OS, LFS, and NRM between the two groups in patients who achieved and remained complete molecular response within 3 months (s3CMR). In patients who did not reach s3CMR, the 3‐year cumulative relapse rate in the ASCT group was significantly higher than that of the MSD‐HSCT group. Therefore, ASCT is an attractive option for patients with Ph^+^ ALL, especially for those with s3CMR maintained up to transplantation^30^ (Figure 3).

**Our recommendations**

- ASCT is recommended for favorable‐risk AML patients in CR1 with negative MRD and an attractive option for intermediate‐risk patients in CR1. In addition, there is a role for ASCT in CR2 patients with APL to achieve a cure.
- ASCT is an attractive option for patients with Ph^+^ ALL, especially for those sustaining s3CMR, maintained up to transplantation.

## 4 PRETRANSPLANT TREATMENT

Pretransplant treatment includes induction and consolidation chemotherapy, which are administered according to the National Comprehensive Cancer Network (NCCN) guidelines.^31^, ^32^ Although prospective randomized trials are missing, available data suggested that patients with AML in CR1 who were going to be treated with ASCT probably benefited from receiving at least one cycle of consolidation before transplant.^33^ One recent study investigated the impact of different pretransplant treatments for ASCT of AML patients ≥60 years. The patients who received I/HDAC (a minimum of one cycle of cytarabine ≥1 g/m^2^ during 3 days at least) did not achieve better outcome than the group receiving 1 + 5 regimen (a minimum of one cycle of anthracycline during 1 or 2 days in association with standard‐dose cytarabine during 5 days [LDAC; ≤200 mg/m^2^]). The posttransplant outcome was not significantly different between the two groups after ASCT (5‐year NRM: 0% vs 10% for patients treated by I/HDAC and 1 + 5 before the transplant, respectively, without significative difference [*P* = .37]). This study showed that pretransplant chemotherapy intensification is not associated with significantly better outcome compared with standard‐dose chemotherapy in elderly patients.^34^ In our study, standard induction chemotherapy given to AML patients included DA (daunorubicin + Ara‐C), MA (mitoxantrone + Ara‐C) or HA (homoharringtonine + Ara‐C). Patients who reached CR were treated with two courses of intensive treatment (MTZ/DNR/demethoxydaunorubicin + medium/high‐dose cytarabine) and two courses of consolidation treatment (standard DA, MA, or HA), and then HSCs were mobilized for storage. All patients newly diagnosed with ALL at IH were given a standard induction regimen: VDCP ± L for a 28‐day cycle (vincristine 1.4 mg/m^2^/d, maximum 2 mg/d, days 1, 8, 15, and 22; daunorubicin 45 mg/m^2^/d, days 1‐3 and 15‐17; cyclophosphamide 750 mg/m^2^/d, days 1 and 8; prednisone 1 mg/m^2^/d days 1‐28; with or without l‐asparaginase 6000 U/m^2^/d, days 5, 8, 11, 15, 18, and 22). After CR was achieved, consolidation chemotherapy was administered, which included several regimens such as high‐dose methotrexate (2 g/m^2^/d, day 1), CAM (cyclophosphamide: 750 mg/m^2^/d, days 1 and 15; arabinoside cytarabine: 200 mg/m^2^/d, days 1‐3 and 8‐10; 6‐mercaptopurine: 60 mg/m^2^/d, generally 100 mg/d, p.o., days 1‐7), dexamethasone (DOAME: 0.15 mg/m^2^/d, days 1‐5; vincristine (VCR) 1.4 mg/m^2^/d, maximum 2 mg/d, day 1; Ara C 2 g/m^2^/d, days 1‐3; mitoxantrone 8 mg/m^2^/d, days 2 and 3; etoposide, 0.1 g/d, days 3‐5).^26^, ^29^ After one or two consolidation courses, stem cell mobilization can be conducted. A total of three to four cycles of consolidation chemotherapy should be applied before ASCT. Prophylaxis of central nervous system leukemia (CNSL) is necessary in ALL, so intrathecal chemotherapy (methotrexate, cytarabine, and corticosteroids) is typically given throughout the entire course of treatment. At least 6 cycles of intrathecal chemotherapy should be conducted before ASCT in ALL,^26^, ^29^ and at IH, three to four cycles of intrathecal chemotherapy are given before ASCT for AML patients.

**Our recommendations**

- The standard induction chemotherapy including DA, MA, or HA should be given to AML patients. Patients who reached CR should be treated with two courses of intensive treatment and two courses of consolidation treatment, after which HSCs should be mobilized for storage.
- All patients newly diagnosed with ALL at IH were given a standard induction regimen VDCLP (vincristine + daunorubicin + cyclophosphamide + L‐asparaginase + prednisone). After achieving CR, consolidation chemotherapy was administered, which included several regimens such as high‐dose MTX, CAM, DOAME, and so on.
- A total of three to four cycles of consolidation chemotherapy should be applied before ASCT.
- Prophylaxis of CNSL is necessary in ALL and intrathecal chemotherapy is typically given throughout the entire course of therapy.
- At least six courses of intrathecal chemotherapy should be given before ASCT for ALL patients.

## 5 STEM CELL MOBILIZATION AND COLLECTION

At IH, most stem cells are harvested from peripheral blood after intensive chemotherapy followed by granulocyte‐colony stimulating factor (G‐CSF).^29^ G‐CSF is given at a dose of 10 μg/kg per day for 7 to 10 days after chemotherapy. Peripheral blood stem cells are collected when the white blood cell count rises to 5 to 10 × 10^9^/L, which is usually achieved at 5 or 6 days after the administration of G‐CSF.^35^ For patients who failed in the first mobilization (CD34^+^ cells <1 × 10^6^/kg in 2 consecutive collection days), another chemotherapy regimen is used to remobilize or collect stem cells from BM as a supplement. There were reports which showed that, Plerixafor, a CXCR4 antagonist, enhanced the engraftment of healthy donor stem cells. Furthermore, plerixafor mobilized and sensitized leukemia cells, which was not suitable for stem cell mobilization in AL patients undergoing ASCT.^36^

The ideal collection time is closely related to the patient's age, previous chemotherapy scheme, mobilization plan, and so on. Therefore, each patient needs to be tested to determine the best collection time. Usually, three factors are considered to determine whether the time is suitable for collection, namely the white blood cell count, the peripheral blood CD34^+^ cell count and the peripheral blood hematopoietic progenitor cell number. However, with the progress of technology and the establishment of detection standardization, the number of CD34^+^ cells in peripheral blood before collection proved to be the most accurate predictor of successful collection.

**Our recommendations**

- G‐CSF should be given at a dose of 10 μg/kg per day for 7 to 10 days after chemotherapy. Peripheral blood stem cells should be collected when the white blood cell count rises to 5 to 10 × 10^9^/L.

## 6 CONDITIONING REGIMENS

At present, total body irradiation (TBI)/cyclophosphamide (Cy) or busulfan (Bu)/Cy are still the two most widely used classic conditioning regimens for ASCT in AL patients. These regimens were originally developed for ASCT and have high immunosuppressive activity, which may not be necessary in the autologous setting. Therefore, researchers have recently attempted to employ novel agents in the conditioning regimens, for example melphalan (Mel), which exhibited stronger antileukemia effects with acceptable or lower hematologic toxicity. Sakaguchi et al retrospectively analyzed the clinical outcomes of 220 AML children who received ASCT after various conditioning regimens. Bu/Cy ± etoposide or Bu/Mel regimens were significantly superior over other Bu‐based and TBI‐based regimens.^37^ Gorin et al also reported 853 patients with AML who underwent ASCT in CR1. Those patients who received a conditioning regimen with Bu/Mel exhibited a lower relapse risk (RR), better LFS and OS than those who were conditioned with BuCy but NRM was similar in the two groups.^38^ In a subsequent study, they further analyzed 1649 adult patients with primary AML and available cytogenetics, autografted from CR1. In the poor risk group, 176 patients received Bu/Cy and 62 patients received Bu/Mel. Bu/Mel was associated with a lower RR at 5 years (53% vs 69%, *P* = .002), a better LFS (42% vs 25%, *P* = .002) and a better OS (54% vs 36%, *P* = .02). However, in the nonpoor risk group, in which 961 patients received Bu/Cy and 450 patients received Bu/Mel, the 5‐year RR (50% vs 47%), LFS (45% vs 48%), and OS (56% vs 60%) was not significantly different. The authors concluded that Bu/Mel was the preferable conditioning regimen for poor‐risk leukemic patients.^39^ In summary, Bu/Mel may be a feasible conditioning regimen for ASCT in patients with AML.

In addition, other conditioning regimens have also been tried. Hong et al believed that idarubicin (IDA)/Bu (I‐Bu) may be a feasible conditioning regimen for patients with AML who received ASCT, and they retrospectively analyzed the outcomes of 32 patients with AML who were performed ASCT in CR1, with an I‐Bu conditioning regimen (IDA 20 mg/m^2^/d × 3 d; Bu 3.2 mg/m^2^/d × 4 days). Of these 32 patients, 31 patients achieved hematopoietic reconstitution; at a median follow‐up of 30 months, 24 patients were still alive and 20 patients continued in CR; the 2‐year RR was 40%.^40^

In ASCT, TBI/Cy as pretransplant regimens is associated with a lower relapse incidence and a higher LFS in ALL than Bu/Cy, which demonstrates that TBI is particularly necessary for ASCT in patients with ALL.^41^ In a study at IH, fludarabine (Flu 30 mg/m^2^/d × 3 days) and cytarabine (Ara‐c 2 g/m^2^/d × 3 days) were added to two classical conditioning regimens to treat 27 patients with ALL receiving ASCT to reduce RR. Hematopoietic reconstitution was achieved in all patients except for one patient who died early. The median time of neutrophil and platelet reconstitution was 11 days^9^, ^10^, ^11^, ^12^, ^13^, ^14^, ^15^, ^16^, ^17^, ^18^, ^19^ and 16 days^10^, ^11^, ^12^, ^13^, ^14^, ^15^, ^16^, ^17^, ^18^, ^19^, ^20^, ^21^, ^22^, ^23^, ^24^, ^25^, ^26^, ^27^, ^28^, ^29^, ^30^, ^31^, ^32^, ^33^, ^34^, ^35^, ^36^, ^37^, ^38^, ^39^, ^40^, ^41^, ^42^, ^43^, ^44^, ^45^, ^46^, ^47^, ^48^, ^49^, ^50^, ^51^, ^52^, ^53^ days, respectively. No serious adverse events occurred during the conditioning, and the 4‐year overall RR, transplant‐related mortality (TRM) and DFS were 38.8%, 9.3%, and 60.8%, respectively.^42^ Thus, the modified standard regimen of patients pretreated by Flu/Ara‐c is an effective and safe approach for ALL patients undergoing ASCT.

**Our recommendations**

- TBI/cyclophosphamide (Cy) or Bu/Cy are still the two most widely used classic conditioning regimens. TBI is particularly necessary for ALL ASCT. The modified standard regimen in which Flu/Ara‐c was added to two classical conditioning regimens is an effective and safe approach.

## 7 MNC/CD34^+^ CELL COUNT

The number of MNC/CD34^+^ cells in the graft is the key factor affecting hematopoietic reconstitution and outcomes after ASCT. Low CD34^+^ cell counts (<2.0 × 10^6^/kg) are not conducive to engraftment. Villalon et al analyzed the factors affecting engraftment of 190 patients with autologous peripheral blood progenitor cell transplantation and found that an infusion of CD34^+^ cells >2.0 × 10^6^/kg resulted in significantly shorter recovery times.^43^ Grubovic et al also demonstrated that neutrophil recovery in AML patients was significantly influenced by transfusion support, according to the number of transplanted CD34^+^ cells and MNC.^44^ However, higher CD34^+^ cell counts (>7 × 10^6^/kg) are related to higher RR, which might be caused by leukemia residual in the autograft.^45^, ^46^ Gorin et al. analyzed 772 patients with ASCT in CR1 and multivariate analysis showed that relapse was more likely in patients who was infused the highest CD34^+^ cell counts (7.16 × 10^6^/kg, *P* = .005).^47^ In a randomized phase 3 AML‐10 trial of European Organization for Research and Treatment of Cancer (EORTC) and Gruppo Italiano Malattie Ematologiche dell'Adulto (GIMEMA) in 292 patients with AML in CR1, it showed that higher number of mobilized CD34^+^ cells were associated with higher RR irrespective of treatment with ASCT or autologous BMT.^45^ At IH, the median number of infused CD34^+^ cells was approximately 2 to 3 × 10^6^/kg, and we also found that CD34^+^ cells in graft >3.8 × 10^6^/kg were a poor prognostic factor of DFS for patients with B‐ALL in ASCT (*P* = .021).^25^, ^27^, ^29^

**Our recommendations**

- The median number of infused CD34^+^ cells should be approximately 2 ~ 3 × 10^6^/kg.

## 8 MAINTENANCE THERAPY AFTER ASCT

Relapse is still the main reason for the failure of ASCT in AL patients, especially ALL patients, so it is necessary to explore the protocols of maintenance treatment after ASCT to reduce the relapse rate and improve the curative effect. At present, the application of maintenance therapy after ASCT is mainly given to patients with ALL, and maintenance therapy at IH for ALL after ASCT usually includes maintenance chemotherapy and immunotherapy (including interleukin‐2 [IL‐2] and interferon‐α).^26^, ^29^, ^48^, ^49^, ^50^, ^51^, ^52^ In addition, for Ph^+^ ALL patients, tyrosine kinase inhibitor (TKI) is also in the list of maintenance regimens.^52^, ^53^ In one study, posttransplant maintenance was applied in 29 cases with the use of either imatinib (n = 25) or dasatinib (n = 4).^52^ In another study, posttransplant maintenance was applied after ASCT with the use of imatinib, nilotinib, or dasatinib.^30^ Wang et al. analyzed 36 patients with ASCT at IH from July 1987 to December 2002. Among them, 11 patients only received alternating maintenance chemotherapy with VP (vincristine 2 mg/d, days 1 and 8; prednisone 30‐40 mg/d, days 1‐14) and MM (6‐mercaptopurine 50‐100 mg/d, days 1‐14; methotrexate 20‐40 mg/d, days 1 and 8) regimens, 5 patients received intermittent IL‐2 (200 000‐400 000 U/d, days 1‐7) immunotherapy, and 8 patients received chemotherapy maintenance combined with IL‐2. Finally, 2 of 24 patients who received maintenance therapy developed TRM, and the RR and 3‐year DFS was 28.9% and 55.7%, respectively.^54^ In a later study, we expanded the sample size to include 70 ALL ASCT patients, of which 52 patients received maintenance chemotherapy after transplantation. We found that the 3‐year DFS of the maintenance treatment group and the nonmaintenance treatment group was 55.12% and 33.33%, respectively (*P* = .050).^50^ Powles et al reported 77 adult ALL patients who underwent ASCT and found that maintenance therapy after ASCT in improved therapeutic efficacy with 42% RR and 53% OS at 10 year follow‐up,^55^ a finding similar to our data. Doubek et al. also found that compared with chemotherapy alone, ASCT combined with maintenance therapy produced longer OS and DFS in both standard‐risk or high‐risk patients.^56^ The role of ASCT in AML with or without hematopoietic graft purging is still uncertain due to persistent high‐risk of post‐transplantation relapse. Thus, standard chemotherapeutic strategies have been tested but are not widely used for AML after ASCT.^57^

**Our recommendations**

- We recommend maintenance chemotherapy for ALL after ASCT with VP (vincristine 2 mg/d, days 1 and 8; prednisone 30‐40 mg/d, days 1‐14) and MM (6‐mercaptopurine 50 to 100 mg/d, days 1‐14; methotrexate 20‐40 mg/d, days 1 and 8) and immunotherapy (including IL‐2 and interferon‐α). In Ph^+^ ALL patients, TKI was also in the list of maintenance regimens for 2 years after ASCT.

## 9 FUTURE DIRECTIONS AND IMPLICATIONS FOR OTHER STEM CELL THERAPIES

**FIGURE 4:**
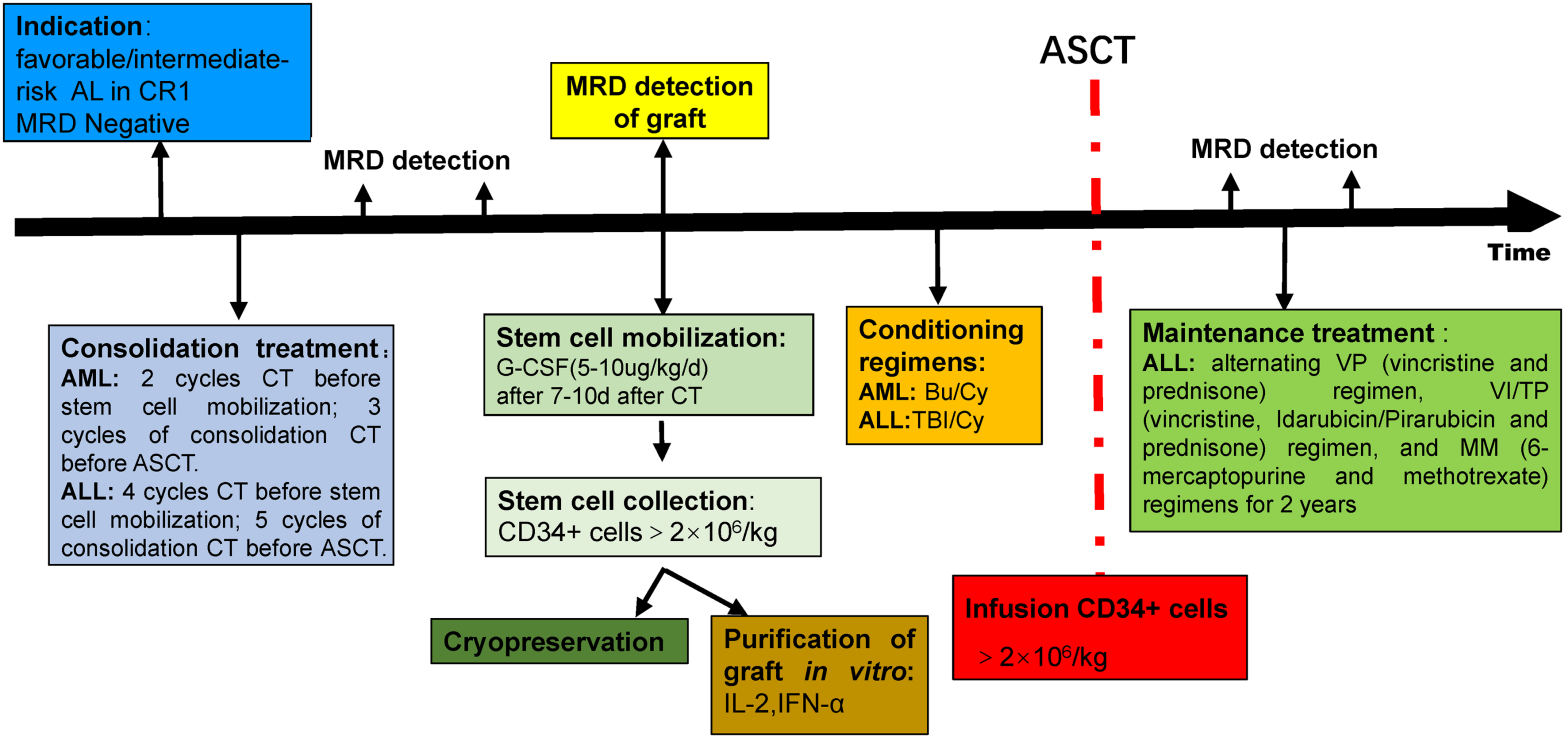
Schema for autologous hematopoietic stem cell transplantation (ASCT) in patients with acute leukemia (AL) in Institute of Hematology and Blood Diseases Hospital of Chinese Academy of Medical Sciences (CAMS) Abbreviations: AL, acute leukemia; ALL, acute lymphoblastic leukemia; AML, acute myeloid leukemia; ASCT, autologous hematopoietic stem cell transplantation; CR, complete remission; CT, chemotherapy

In summary, since the first case of ASCT in China was performed by Professor Wenwei Yan at IH of CAMS in 1986, it has taken almost four decades to optimize ASCT for AL and form the protocols of ASCT at IH of CAMS (Figure 4). There are both opportunities and challenges in the new situation for ASCT. Breakthrough technologies such as gene editing, next‐generation sequencing (NGS) and targeted therapies will be powerful weapons to optimize the transplantation procedure. When ASCT is to be performed, MRD status should be taken into consideration, so improving the sensitivity and specificity of MRD detection by NGS absolutely favors patients who are qualified for ASCT. MRD‐negative patients with AL in CR1 (or possibly CR2) are worthy of note in more multicenter, random and prospective trials. In addition, new conditioning regimens also display tremendous potential for better LFS and OS, particularly Bu/Mel, which is associated with superior antileukemia effects.^38^, ^39^ A series of targeted drugs or chimeric Ag receptor T‐cell immunotherapy are currently undergoing clinical trials for AML and ALL. Any of these therapies could combine with ASCT to achieve a satisfying prognosis.^58^

## AUTHOR CONTRIBUTIONS

A.P. wrote the first draft of the manuscript. Y.H., B.S., Y.Z., E.J., S.F. and M.H. all contributed to and approved the final version.

## CONFLICT OF INTEREST

The authors declared no potential conflicts of interest.

## Data Availability

Data sharing is not applicable to this article as no new data were created or analyzed in this study.
